# Cellular Memory of HipA-Induced Growth Arrest: The Length of Cell Growth Arrest Becomes Shorter for Each Successive Induction

**DOI:** 10.3390/microorganisms9122594

**Published:** 2021-12-15

**Authors:** Chun-Yi Lin, Sanya Hamini, Peter Robert Tupa, Hisako Masuda

**Affiliations:** 1 Department of Biochemistry and Molecular Biology, Rutgers University, 675 Hoes Lane, Piscataway, NJ 08854, USA; cylin520@ntu.edu.tw; 2 School of Sciences, Indiana University Kokomo, 2300 S Washington St., Kokomo, IN 46902, USA; sanyarangwala@gmail.com (S.H.); ptupa@iuk.edu (P.R.T.)

**Keywords:** HipA, toxin–antitoxin system, persister, growth arrest, cellular memory

## Abstract

Toxin–antitoxin (TA) systems are genetic modules found commonly in bacterial genomes. HipA is a toxin protein encoded from the *hipBA* TA system in the genome of *Escherichia coli.* Ectopic expression of *hipA* induces cell growth arrest. Unlike the cell growth arrest caused by other TA toxins, cells resume growth from the HipA-induced cell growth arrest phase after a defined period of time. In this article, we describe the change in the length of growth arrest while cells undergo repeated cycles of *hipA* induction, growth arrest and regrowth phases. In the multiple conditions tested, we observed that the length of growth arrest became successively shorter for each round of induction. We verified that this was not due to the appearance of HipA-resistant mutants. Additionally, we identified conditions, such as the growth phase of the starting culture and growth vessels, that alter the length of growth arrest. Our results showed that the length of HipA-induced growth arrest was dependent on environmental factors—in particular, the past growth environment of cells, such as a previous *hipA* induction. These effects lasted even after multiple rounds of cell divisions, indicating the presence of cellular “memory” that impacts cells’ response to HipA-induced toxicity.

## 1. Introduction

Actively growing populations of bacteria harbor a subpopulation of dormant cells [1,2]. These cells are insensitive to the action of antibiotics and other stresses [3,4]. Once stress is removed, dormant cells can resume growth, and the population will again become a mixture of actively growing and dormant cells. This phenomenon of forming a persistent subpopulation is distinctive from antibiotic resistance, which involves genetic changes. Persister cells are of clinical importance, as these cells are responsible for recurring infections [3]. Recent studies suggest that reduction of the growth rate via inhibition of a core cellular activity can create a non-growing, persistent subpopulation [5,6].

HipA is a toxin of a TA system on the *E. coli* genome [7]. TA systems are small genetic modules that are ubiquitously found in bacterial genomes [8,9]. TA modules are composed of a pair of co-transcribed toxin and antitoxin genes [8]. In type II TA systems, toxin proteins target a variety of essential cellular processes, including protein translation, DNA replication and cell division [10,11,12,13,14,15]. Antitoxins sequester toxins from their targets via the formation of a stable complex until the stress-inducible proteases, such as Lon, preferentially degrade the antitoxin proteins, freeing the toxin proteins to exert their inhibitory activity [16,17]. A gain-of-function mutant allele of *hipA* (*hipA7*) was first identified as a genetic locus responsible for a high proportion of persister cells [7].

HipA is a kinase [18,19]. HipA7 is encoded from a mutant allele (*hipA7*) harboring two amino acid substitutions [20]. HipA7 encoded endogenously from the chromosome is shown to phosphorylate Glu-tRNA-ligase (GltX) [21]. Phosphorylation inhibits GltX activity, which increases the amount of uncharged tRNA(Glu) [19]. This, in turn, induces a stringent response (i.e., increase in the ppGpp levels via RelA/SpoT) [19], leading to the inhibition of transcription, translation, replication and cell wall synthesis. As a result, cells halt growth and become dormant [19].

When wildtype *hipA* was expressed endogenously from the chromosome, the phosphorylation of GltX was not observed [21]. It is proposed that this is because HipA forms a complex with co-expressed antitoxin HipB. When wildtype *hipA* was expressed at a higher level via ectopic expression, GltX phosphorylation was observed [19,21]. The results demonstrate that a higher level of free HipA than HipB is necessary to inhibit its target(s).

A direct tie between *hipA* expression and growth arrest has been demonstrated. HipA overexpression halts cells growth, which can be reversed by the expression of HipB [22]. A single-cell study of *hipA* revealed that the concentration of HipA needs to exceed a threshold to induce growth arrest [23]. Stochastic variations in the HipA level within a population creates a mixture of dormant and actively growing cells. The authors also showed that the length of dormancy depends on the level of HipA. Furthermore, the overexpression of GltX can reverse the *hipA*-induced toxicity, validating the link between *hipA*-induced cell growth arrest and inhibition of GltX [18,19].

The overexpression of *hipA* in cells grown in liquid medium exhibit a unique growth curve [22]. The growth arrest is transient despite the continuous presence of inducers. Following a few hours of arrest, cells resume growth even without the ectopic induction of HipB [22]. The same growth pattern has not been seen for other TA toxins, such as YoeB, YeeV and CptA [11,24,25]. In such cases, growth arrest continues until cells become no longer viable.

While memory or learning is traditionally linked to multicellular organisms, recent studies suggest that these behaviors exist even in single-cellular organisms, such as bacteria. Bacterial regulatory networks exhibit complex dynamics and multi-stable behaviors, which are the hallmark of memory in other organisms [26,27]. Moreover, bacterial cells are capable of exhibiting variable responses to their current conditions based on past history [26,28,29,30,31]. For example, cells respond faster to a phosphate limitation or switch in carbon sources when the same signal has been perceived in the past [26,28]. Sporulation initiation and extracellular protease expression were also altered by past growth history [29]. The timing of an individual cell’s entry into the stationary phase affects each the cell’s subsequent timing for resuming growth [30]. This effect lasts for several days and creates a heterogeneous population with a mixture of active and dormant cells. Long-lasting memory has also been observed in multiple other bacterial strains, including *Staphylococcus*, *Bacillus*, *Acinetobacter* and *Salmonella* [31].

In this article, we describe our discovery that the length of HipA-induced growth arrest varies depending on the previous exposure to HipA expression in *E. coli*. When HipA was expressed ectopically for the first time, the growth arrest lasted 3–5 h, depending on the growth conditions used. When *hipA* expression was induced again following the regrowth phase, the growth arrest either did not occur or occurred but for a much shorter period than the previous induction phase. The same trend was observed for three rounds of induction. We also identified additional factors that influence the length of growth arrest caused by HipA toxicity. Our study demonstrated that the length of growth arrest by HipA-induced toxicity appears to be dependent on the past growth conditions, including but not limited to past HipA expression.

## 2. Materials and Methods

### 2.1. Bacterial Strain, Culture Conditions and Vectors

For growth analysis, *E. coli* BW25113 (*F^−^, rrnB, ΔlacZ4787, HsdR514, Δ(araBAD)567* and *Δ(rhaBAD)568, rph-1*) was used [32]. Cells were grown in M9 medium supplemented with 0.9% casamino acids, 1-mM thiamine and 0.5% glycerol. Thirty micrograms per milliliter of chloramphenicol were added to the medium for cells harboring plasmid derived from pBAD33 (M9-Cm) [33]. The coding sequence of *hipA* was cloned into pBAD33, and it was kindly gifted from Dr. Masayori Inouye’s laboratory at Rutgers University for this project. The plasmid was referred to as pBAD33_*hipA* in this manuscript. The expression of *hipA* was induced by the addition of 0.2% arabinose. De-ionized water was used in all experiments. Plasmid DNA was extracted with the mini lysis procedure [34].

### 2.2. Measurement of Cell Growth

**Figure 1:**
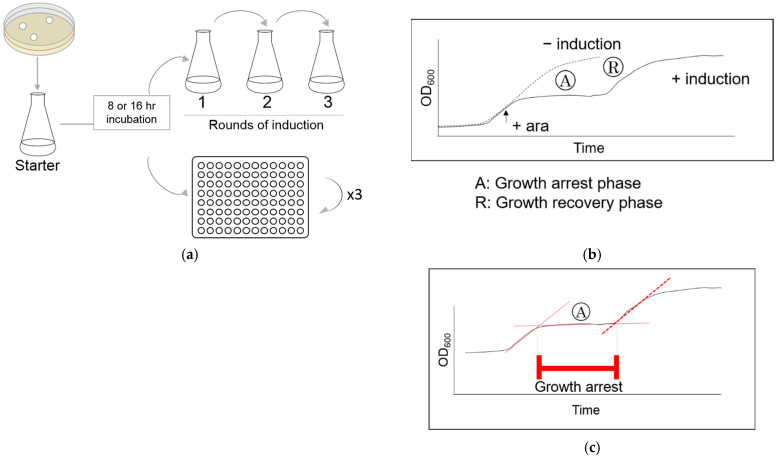
Experimental scheme and data analysis. (**a**) Starter culture was created by inoculating the bacteria from the solid medium. The first experimental cultures were created by subculturing the starter culture at either the early (8 h) or late (16 h) stationary phase. HipA expression was induced in flasks 1–3. (**b**) The typical shape of the growth curve with (+induction) and without (−induction) HipA induction. Arabinose was added at the mid-exponential phase (+ara). The growth arrest phase (A) was followed by a growth recovery phase (R). The growth curve without an inducer is shown as a dotted line. (**c**) The length of the growth arrest was calculated based on the intersect of the linear regression lines.

Cells were grown in Erlenmeyer flasks at 37 °C either to the early (8 h) or late (16 h) stationary phase (Figure 1a). Cells were transferred to a fresh medium, then grown in the flask or on a microplate at 37 °C with constant agitation at 200 rpm or at the highest setting, respectively. HipA expression was induced with 0.2% arabinose when the optical density at 600 nm (OD_600_) reached 0.15 or 0.05 for the experiments with flasks or microtiter plates, respectively (Figure 1b + ara). After the induction for 15 min, cells were washed by fresh medium three times before they were transferred to a new flask or microplate. All experiments were performed in triplicate.

Cell growth was monitored by measuring the absorbance at 600 nm with a spectrophotometer (Jenway 6715, Cole-Parmer, Eaton Socon, UK) when grown in a flask. Changes in the OD_600_ in the microplates were continuously monitored by AccuSkan Microplate Photometer (Thermo Fisher Scientific, Waltham, MA, USA). Phases of the growth curve were defined as shown in Figure 1b (“A”: growth arrest phase and “R”: growth recovery phase).

### 2.3. Induction of HipA during the Rapid Growth Recovery Phase

The cells were removed from the middle of the growth recovery phase (Figure 1b—phase “R”). The OD_600_ was adjusted to be approximately 0.05 with fresh medium and separated into two sets of three Erlenmeyer flasks. Arabinose was added to one of the sets. For the experiments with the microtiter plate, half of the wells were treated with arabinose, and the rest served as a control. All samples were grown for 15 min at 37 °C with arabinose, then washed three times with fresh medium. The cells were then transferred to a flask or to microplate, and their growth was monitored. The same process was repeated to study the growth arrest during the third round of induction.

### 2.4. Computational and Statistical Analysis

The length of the growth arrest phase (Figure 1b—phase “A”) was calculated by finding the intersect of two linear regression lines (Figure 1c). The beginning of the growth arrest was defined as the intersect of the linear regression lines for the exponential growth phase and for the growth arrest phase. The end of growth arrest phase was defined as the intersect of the linear regression lines for the growth arrest phase and growth recovery phase. The differences in the x-values were defined as “the length of the growth arrest”.

An in-house bash script was used to process the optical density data. The growth curve was constructed using ggplot2 in R [35]. The differences in the lengths of growth arrest were analyzed by a *t*-test.

### 2.5. Test of Genetic Mutation

**Figure 2:**
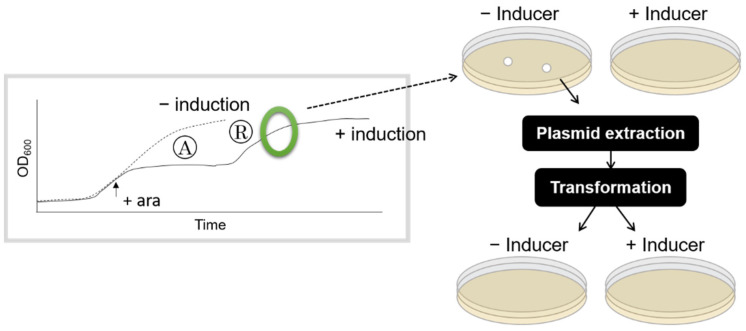
Flow chart describing how the absence of genetic change was verified. Green circle indicates when the cells were collected. Cells were replicas plated onto a M9-Cm medium and a M9-Cm medium supplemented with arabinose to induce *hipA* expression. Plasmids were also extracted from these cells, transformed into a fresh *E. coli* culture and plated onto two types of agar plates consisting of the two different media described above.

Cells from the recovery phase after each round of HipA induction were collected and plated on solid M9 medium supplemented with chloramphenicol. Fifty colonies from each plate were transferred to a solid M9 chloramphenicol medium (“−induction” in Figure 2) and to a M9 chloramphenicol medium containing 0.2% arabinose (“+induction” in Figure 2). The formation of a colony after incubation at 37 °C for overnight was recorded. The plasmid DNA was extracted from each clone and retransformed to test the ability to induce growth arrest on the solid medium.

## 3. Results

### 3.1. HipA-Induced Growth Arrest Phase Shortens in Each Round of Induction in Erlenmeyer Flasks

**Figure 3:**
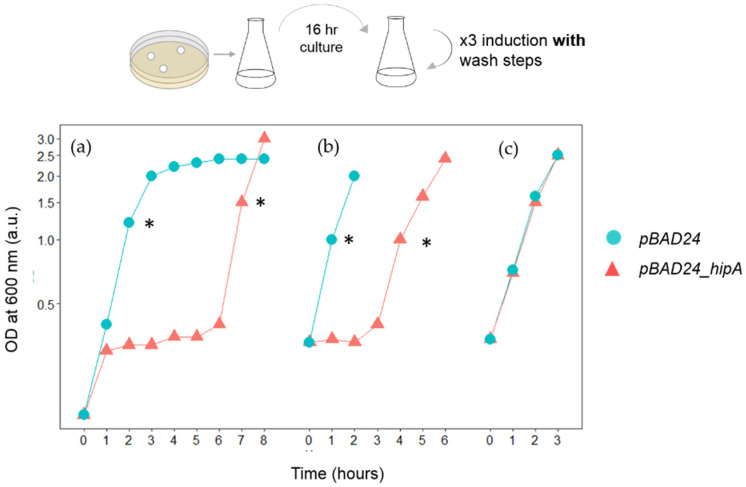
The growth curve of three consecutive inductions of HipA expression in Erlenmeyer flasks. A starter culture grown for 16 h was subcultured to create the first experimental sample. *E. coli* BW25113 cells carrying empty *pBAD24* (circle) or *pBAD24_hipA* (triangle) were grown in Erlenmeyer flasks at constant agitation. T = 0 in each figure indicates the time after cells were incubated with 0.2% arabinose for 15 min, washed three times and returned to the flask. The * symbols indicate when cells were removed, the OD_600_ was adjusted, the *hipA* expression was induced for 15 min, the inducer was removed and monitoring of the OD change started for the next round. Growth curves for the first (**a**), second (**b**) and third (**c**) rounds of inductions are shown.

The growth curve of *E. coli* BW25113 carrying pBAD33_*hipA* grown in an Erlenmeyer flask with and without the induction of *hipA* induction is shown in Figure 3a. The expression of *hipA* was induced by incubation with 0.2% arabinose for 15 min, and then, arabinose was removed by centrifugation and resuspension with fresh medium. The first data point indicates the time when the cells were returned to the flask following the removal of arabinose. The increase in OD_600_ halted after 1 h of incubation. The growth arrest lasted approximately until 6 h post-induction (Figure 3a). The cells then resumed growth at an equivalent rate as pre-induction (named the “growth recovery phase”). Rapidly growing cells from the recovery phase were immediately transferred to a new flask, the OD_600_ was adjusted to 0.35 with fresh M9 medium, the expression of *hipA* was induced again for 15 min, the cells were collected by centrifugation and the cell pellet was resuspended in fresh medium. This wash step was repeated three times to ensure the removal of arabinose from the medium. An immediate cell growth arrest was observed (pink line in Figure 3b). This was a stark contrast to the control culture containing empty plasmid, which started a rapid growth immediately (blue line in Figure 3b). The growth arrest in the induced culture only lasted for 3 h, which was significantly shorter than the duration of growth arrest during the first round of induction (5 h). When *hipA* was induced for the third time, growth arrest was no longer observed (Figure 3c).

### 3.2. Shorter Growth Arrest Phase Is Not Due to an Increase in HipA-Resistant Mutants

We then tested whether a shorter growth arrest was due to a rise in a subpopulation of mutants resistant to *hipA*-induced toxicity (Figure 2). Fifty independent clones from the regrowth phases were tested for *hipA*-induced cell growth inhibition on M9 agar plates containing arabinose. For the culture in the third round of induction, the cells were collected after reaching the stationary phase. As summarized in Table 1, 100% of the surveyed cells retained a sensitivity to *hipA*-induced toxicity. No growth was observed on arabinose containing solid media. Even the clones that were collected from the culture that underwent three rounds of induction did not include HipA-resistant clones. To further confirm that there was no genetic change in the plasmids that led to the observed phenotype, the plasmids were extracted from the clones mentioned above and retransformed into the fresh wildtype BW25113 cells. *hipA*-induced toxicity was again detected, as no colony was formed. These results confirmed that there was no genetic change in *hipA*-pBAD33 that abolished *hipA*-induced cell growth inhibition.

### 3.3. Quantitative Analysis of HipA-Induced Growth Arrest in Microplates

**Figure 4:**
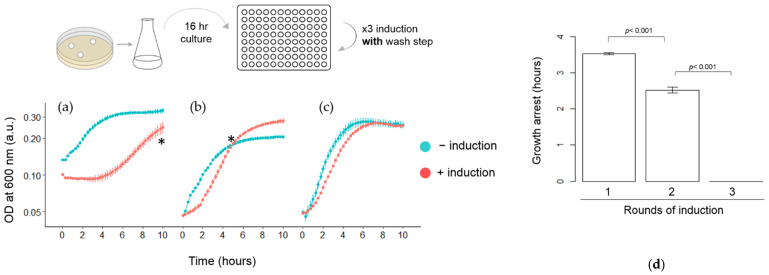
The growth curve of three consecutive inductions of HipA expression on microplates. *E. coli* BW25113 cells carrying *pBAD24_hipA* were grown on microplates with constant agitation. A starter culture grown for 16 h was used to inoculate the first culture. The pink and blue points represent data for a culture with and without a 0.2% arabinose addition, respectively. T = 0 in each figure indicates the time after cells were incubated with 0.2% arabinose for 15 min, washed three times and returned to the microplate. (**a**) Growth curve after the first induction. The * symbol indicates when the samples from the induced culture were removed to initiate the induction, washed and monitoring the growth was started for the second round. The same cells were subcultured to create the “−induced” culture to which arabinose was not added, but the same treatment was implemented as the “+induction” samples. (**b**) Growth after the second induction, (**c**) growth after the third induction and (**d**) a summary of the length of the growth arrest phase in each round. The value above the graph indicates the *p*-value from the *t*-test.

**Figure 5:**
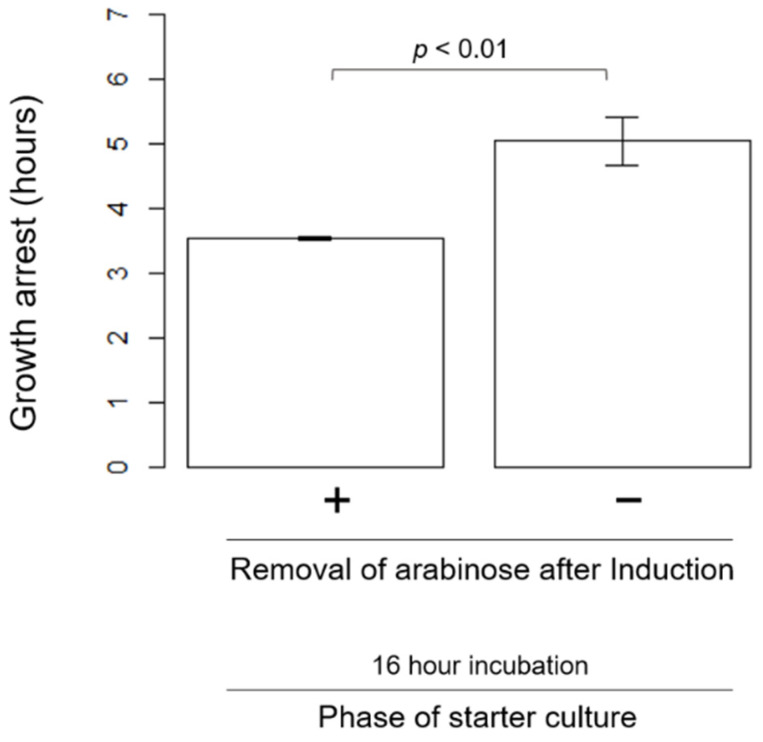
Comparison of the growth arrest phases with and without the removal of arabinose after induction. The value above the graph indicates the *p*-value from the *t*-test. The measurements were performed using microtiter plates.

**Figure 6:**
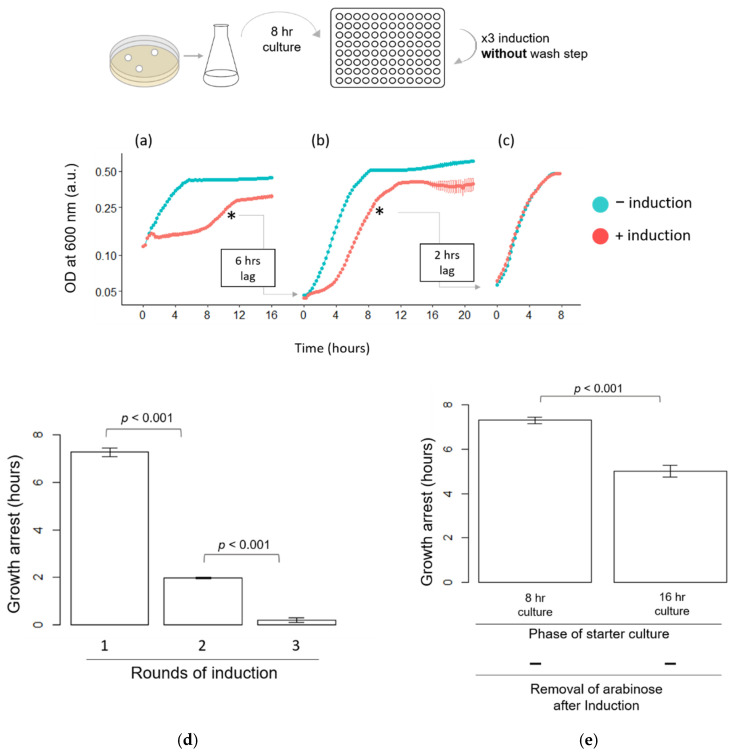
Effects of the growth phase of a starter culture on HipA-induced growth arrest. *E. coli* BW25113 cells carrying *pBAD24_hipA* were grown on a microplate with constant agitation. Pink and blue points represent data for the cultures with and without a 0.2% arabinose addition, respectively. T = 0 in each figure indicates the time when 0.2% arabinose was added. Arabinose was not removed. Cells were grown from the early stationary phase (8 h post-inoculation) starter culture, and HipA was induced one (**a**), two (**b**) and three (**c**) times. (**d**) Summary of the growth arrest phase in each round of induction. (**e**) Comparison of the growth arrest after the first induction of HipA using the starter culture at the early (8 h) or late (16 h) stationary phase. The * symbol indicates when the cells were removed to initiate the culture for the next round. The value above the graph indicates the *p*-value from the *t*-test.

For a more precise comparison of the length of the growth arrest in each round of induction, we performed the following experiments (shown in Figure 4, Figure 5 and Figure 6) using microplates, which allowed continuous monitoring of the growths of multiple cultures. To ensure that the growth arrest was not due to the artifact from the subculturing and washing process, the cells from the regrowth phase were separated into two groups after adjusting the OD_600_: (1) arabinose was added, and (2) arabinose was not added. Otherwise, both groups were processed in the same manner. HipA-induced growth arrest was observed when HipA was induced for the first time (Figure 4a). However, the length of the growth arrest was shorter (3.54 ± 0.07 h; Figure 4a) than when the experiments were performed in flasks (5 h; Figure 3). When previously induced cells were transferred to a fresh medium and regrown for the second and third time without arabinose, they exhibited rapid growth immediately following the subculturing (Figure 4b,c, −induction). In arabinose-treated cultures, the length of growth arrest became shorter for the second induction (2.52 ± 0.21 h; Figure 4b, +induction) than the first round (3.54 ± 0.07 h; Figure 4a, +induction). No obvious growth arrest was observed when *hipA* was induced for the third time (Figure 4c). The comparison of the growth arrest phase (Figure 4d) showed successive reduction. It is also notable that, while an increase in the OD_600_ halted completely after the first induction (Figure 4a; 0–2.5 h), the OD_600_ exhibited a slight increase after the second induction (Figure 4b; 0–2 h).

### 3.4. Other Factors Influencing the Length of HipA-Induced Growth Arrest Phase

We then looked for a factor(s) to alter the length of the growth arrest phase and/or a successive reduction in the arrest phase length. First, we tested whether the continuous induction of HipA changes the cell growth arrest phase. To test this possibility, we conducted the experiments without the removal of arabinose after the 15-min induction. The length of the growth arrest became longer (5.0 ± 0.8 h) than when arabinose was removed after the induction (3.54 ± 0.07 h) (Figure 5). Growth recovery still happened, even in the absence of the inducer removal step.

Next, we examined the effects of the age of the starter culture that was used to inoculate the experimental culture. For experiments shown in Figure 3 and Figure 4, the first experimental culture (culture #1 in the Figure 1a) was created from the starter culture in the late stationary phase (16 h post-inoculation). As shown above, the growth arrest phase lasted 5.0 ± 0.8 h when the inducer was not washed using the late stationary culture. When culture #1 was created from the starter culture at the early stationary phase (approximately 8 h post-inoculation), the length of the growth arrest was 7.2 ± 0.45 h (Figure 6a). This is significantly longer than when the late stationary phase cells were used to create the culture and arabinose was not removed (Figure 5 and Figure 6e). Thus, the age of the starter culture cells used to create the subculture and a lack of removal of the inducer synergistically influenced the length of the growth arrest.

Using this condition, we examined whether successive shortening of the growth arrest phase was observed without washing the inducer. It was notable that the cells from the recovery phase did not exhibit immediate growth. The lag phase lasted for 6 and 2 h after being transferred to a fresh medium after the first and second growth recovery phases, respectively. This was a sharp contrast to the earlier experiments in which arabinose was removed after 15 min of induction (Figure 3 and Figure 4). Under such conditions, the cells initiated growth immediately after the transfer. Despite the presence of the lag phase in the absence of the wash step, when HipA was induced for the second and third time, cell growth arrest and the cell growth resumption were observed (Figure 6b,c, respectively). A continuous decrease in growth arrest was also observed for each successive induction (2.0 ± 0.10 and 0.18 ± 0.25 h, respectively; Figure 6d). These results showed that, when *hipA* was continuously expressed and inoculated with a starter culture at a different growth phase, shortening of the growth arrest could still occur.

## 4. Discussion

In this study, we detected decreases in the duration of growth arrest when HipA was reinduced after the initial growth arrest. Shortening of the growth arrest phase at the subsequent round(s) of induction was observed under different conditions. Several factors were also found to affect the length of arrest, such as the growth environment, age of cells used to prepare the culture and the presence or absence of an inducer removal step.

Our results showed that the loss of sensitivity to HipA was not caused by the emergence of a HipA-resistant subpopulation. Alteration of the plasmids that abolish the expression of HipA was not detected. This strongly supports the notion that the differences in the length of growth arrest are not due to a genetic change but due to phenotypic differences that reflect their past growth conditions.

An earlier single-cell analysis of an HipA-induced population revealed that the HipA expression level was positively correlated with the length of the growth arrest [23]. Our experiments that compared the effects of the wash step after induction corroborated this observation. The unwashed cells exhibited a longer growth arrest than when the inducers were washed after 15 min of induction (Figure 5). The absence of the wash step would allow for a higher and/or longer expression of HipA, which perhaps, in turn, could increase the length of the growth arrest phase.

Regardless of the presence or absence of the inducer removal step, we observed the reduction in the growth arrest phase in the successive rounds of inductions (Figure 4 and Figure 6). This effect lasted longer than one cell cycle. This is reminiscent of other phenomena associated with memory in bacteria (i.e., different phenotypes based on past histories and effects that can last for several generations). Miyaue et al. reported that the cells in a biofilm contained a higher fraction of persisters than in a liquid culture [31]. The fraction of persisters remained high even after a prolonged period following the transfer from the biofilm to the liquid culture.

Various mechanisms for non-genetic memory have been described for single-cellular organisms. Cells in a biofilm exhibit membrane potential-based memory when transiently exposed to light [36]. Potassium ion channels are involved in changing the membrane potential. A faster response to recurring phosphate limitations is mediated by two-component regulatory systems [26]. Signal transduction systems are also implicated in a positive effect of memory in chemotaxis and nitrogen starvation [27,37]. Our group is currently investigating the molecular mechanisms of how cells have “memories” of past HipA-induced growth arrests.

Our study also revealed that growth conditions impact the length of HipA-induced growth arrest. When grown in microplates, the duration of the growth arrest was shorter than when grown in a larger volume in flasks (Figure 3). While we used the same medium, initial cell density and incubation temperature, the mode of agitation and the sample’s volume were different for the Erlenmeyer flasks and microplates. The different effectiveness of agitation and/or the amount of head space would most likely change the level of oxygenation and the diffusion of nutrient and waste materials within the medium.

The growth phase of the cells used to inoculate the experimental culture also influenced the length of the growth arrest during the first induction (Figure 6e). This result implies that, despite cells collectively undergoing multiple-cell divisions and transitions from stationary to lag and then to exponential phases, the cellular states from the starter culture impact the future responses to HipA-induced growth arrest. Similarly, in *Salmonella typhimurium*, the effects of the growth phase of the starter culture (e.g., early or later stationary phases) on the lag phase of the experimental culture have been reported [38].

It remains unclear how cells resume growth after HipA-induced growth arrest even when the inducer is not removed from the medium. A similar study with other TA toxins did not show a similar phenomenon [11,24,25]. One possible mechanism is a specific cellular response to reverse HipA-induced growth arrest. Kaspy et al. observed that the overexpression of GltX, the glutamyl–tRNA synthetase, abolished HipA toxicity [19]. Our preliminary study did not detect a significant increase of *gltX* expression during the growth arrest or recovery phase. However, other proteomic and phosphoproteomic changes may allow cells to resume growth despite the presence of HipA, as HipA is shown to phosphorylate multiple other cellular proteins and itself (autophosphorylation) [21,39]. The autophosphorylation of HipA as a regulatory mechanism has also been reported [40,41].

Since the kinase activity of HipA is critical for growth arrest [40], HipA targets may play a role in growth arrest, as well as growth recovery. A future inquiry of proteins phosphorylated by HipA would also provide insights as to how the growth arrest becomes successively shortened. Our group is currently investigating such proteomic, as well as transcriptomic, differences that impact the growth arrest and its memory.

## Figures and Tables

**Table 1:** Summary of the search for HipA-resistant mutants.

Samples	Cells Exhibiting Arabinose-Inducible Toxicity (*n* = 50)	Plasmids Conferring HipA Toxicity (*n* = 50)
Initial	100%	100%
After the first round of induction and regrowth	100%	100%
After the second round of induction and regrowth	100%	100%
After the third round of induction and regrowth	100%	100%
